# SMASH, a fragmentation and sequencing method for genomic copy number analysis

**DOI:** 10.1101/gr.201491.115

**Published:** 2016-06

**Authors:** Zihua Wang, Peter Andrews, Jude Kendall, Beicong Ma, Inessa Hakker, Linda Rodgers, Michael Ronemus, Michael Wigler, Dan Levy

**Affiliations:** Cold Spring Harbor Laboratory, Cold Spring Harbor, New York 11724, USA

## Abstract

Copy number variants (CNVs) underlie a significant amount of genetic diversity and disease. CNVs can be detected by a number of means, including chromosomal microarray analysis (CMA) and whole-genome sequencing (WGS), but these approaches suffer from either limited resolution (CMA) or are highly expensive for routine screening (both CMA and WGS). As an alternative, we have developed a next-generation sequencing-based method for CNV analysis termed SMASH, for short multiply aggregated sequence homologies. SMASH utilizes random fragmentation of input genomic DNA to create chimeric sequence reads, from which multiple mappable tags can be parsed using maximal almost-unique matches (MAMs). The SMASH tags are then binned and segmented, generating a profile of genomic copy number at the desired resolution. Because fewer reads are necessary relative to WGS to give accurate CNV data, SMASH libraries can be highly multiplexed, allowing large numbers of individuals to be analyzed at low cost. Increased genomic resolution can be achieved by sequencing to higher depth.

Analysis of CNVs on a genomic scale is useful for assessing cancer progression and identifying congenital genetic abnormalities (Hicks et al. 2006; Sebat et al. 2007; Marshall et al. 2008; Xu et al. 2008; Levy et al. 2011; Stadler et al. 2012; Warburton et al. 2014; for review, see Malhotra and Sebat 2012; Weischenfeldt et al. 2013). CNVs are typically identified by microarray hybridization (Iafrate et al. 2004; Sebat et al. 2004) but can also be detected by next-generation sequencing (NGS). This is generally done using algorithms that measure the number of sequence reads mapping to specific regions (Alkan et al. 2009); consequently, the resolution of sequence-based copy number methods depends largely on the number of independent mappings. The current trend in NGS technologies is to increase the number of bases read per unit cost. This is accomplished by increasing the total number of sequence reads per lane of a flow cell, as well as increasing the number of bases within each read. Because the accuracy of copy number methods is driven by the quantity of unique reads, increasing the length of reads does not improve the resolution or decrease the cost of copy number analysis.

Most of the human genome is mapped well by short reads, on the order of 35–40 bp (Supplemental Fig. S1). At the moment, high-throughput sequencers with the greatest per base cost effectiveness are generating paired-end read lengths of 150 bp, well in excess of what suffices for unique mapping. In fact, variability in insert size and “mappability” of paired-end reads suggest that paired-end mapping is a poor choice for read-depth–based copy number analysis of WGS. To take advantage of current (and future) increases in read length and optimally utilize paired-end reads, we have developed SMASH to “pack” multiple independent mappings into every read pair. We accomplish this by breaking genomic DNA into small fragments with a mean length of ∼40 bp. These fragments are joined together into chimeric stretches of DNA with lengths suitable for creating NGS libraries (300–700 bp). SMASH is conceptually similar to serial analysis of gene expression (SAGE) (Velculescu et al. 1995), which utilized the generation of chimeric molecules of short cDNA-derived tags to provide a digital readout of gene expression. SMASH differs in that it (1) requires significantly longer tags than SAGE and its later variants (e.g., SuperSAGE) (Matsumura et al. 2008) due to the complexity of genomic DNA, and (2) utilizes mechanical shearing and/or enzymatic digestion to counteract restriction enzyme bias, creating highly variable fragments of genomic DNA.

The chimeric sequence reads generated by SMASH are processed using a time-efficient, memory-intensive mapping algorithm that performs a conservative partition of the long read into constituent fragments. The fragment maps are utilized in the same manner as read maps in downstream copy number analysis. For 125-bp paired-end reads, whole-genome sequencing (WGS) averages less than one map per read pair, whereas SMASH yields four to five. The quality of SMASH maps, i.e., the nonuniformities introduced by the sample preparation and sequencer and mapping bias, is of the same order as those seen with WGS mapping. Using correction and testing protocols optimized for WGS data, we show that on a map-for-map basis, SMASH generates read-depth copy number data that is virtually equivalent to WGS at a small fraction of the cost.

## Results

### Overview

**Figure 1.**
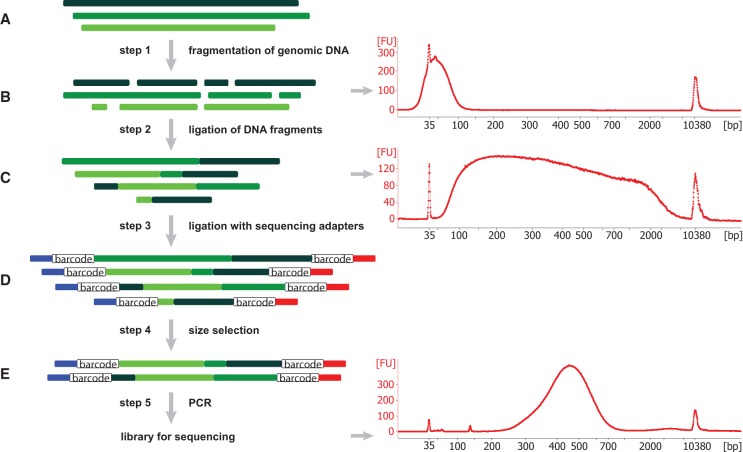
Schematic of the SMASH method and size analysis. (*A*) Three representative genomic DNA molecules, shown in different shades of green, originate from different chromosomes or distant regions of the same chromosome. (*B*) By sonication and restriction enzyme cleavage, these molecules are fragmented into short double-stranded DNA fragments with average length of 40–50 bp, as shown in the Bioanalyzer result at *right*. (*C*) These short DNA pieces are then partially end-repaired and combined into longer stretches of DNA with lengths ranging from 50 bp to 7 kb. Consequently, each resulting chimeric DNA molecule contains short DNA fragments from different locations (shown by varying colors). (*D*) These DNA stretches are ligated to sequencing adaptors containing sample barcodes, shown in blue and red lines, with the open box designating the sample barcodes. (*E*) Size selection is carried out to enrich for DNA fragments in the size range of 250–700 bp, which is confirmed via Bioanalyzer. After final PCR, libraries are ready for sequencing. “FU” in the Bioanalyzer plots refers to relative fluorescence units.

The SMASH protocol (Methods) is illustrated in Figure 1. To obtain short fragments of genomic DNA, we first mechanically shear by sonication and then cut with two restriction endonucleases (REs). We then use bead purification to enrich for the target size range of ∼40 bp (Fig. 1, right). To generate the long chimeric DNA molecules for sequencing, we then end-repair the short fragments and ligate them together. Barcoded sequencing adaptors are attached to the ends of the molecules so multiple libraries can be run together on a single lane. DNA fragments in the optimal size range (300–700 bp) are then selected by bead purification. This protocol is robust and reproducible, typically generating libraries with nearly identical distributions of fragment and chimera lengths (Supplemental Fig. S2). All data utilized in this work were generated using the initial protocol. Subsequently, an improved SMASH protocol (described further in the “alternate fragmentation” section of the Results) has been developed that substitutes digestion by dsDNA fragmentase for sonication and restriction digests, reducing the cost and time of sample preparation (Methods; Supplemental Figs. S3, S4).

**Figure 2.**
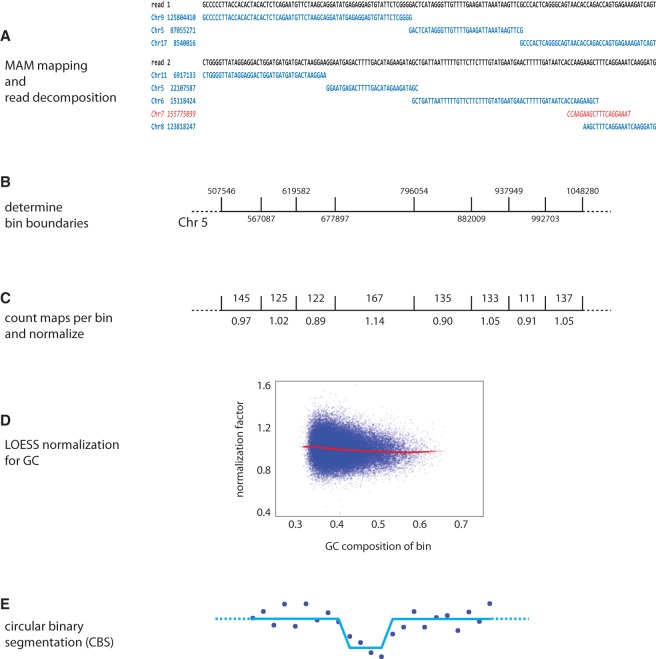
SMASH informatics pipeline. (*A*) The decomposition of a read pair into a set of maximal uniquely mappable fragments is shown. In contrast to the red maps, the blue maps satisfy the 20:4 rule and are considered countable maps. (*B*) Bin boundaries are selected such that each bin has the same number of exact matches from all 50-mers from the reference genome. A representative stretch of Chromosome 5 is displayed. (*C*) The numbers of 20:4 mappable fragments present in each bin are counted, with duplicate reads excluded. The number *above* the bin shows the count of maps, and the number *below* shows the normalized value. (*D*) LOESS normalization is used to adjust bin counts for sample-specific GC bias. (*E*) The data are segmented using circular binary segmentation (CBS) of the GC-normalized data.

To recover mapping information from the chimeric reads, we apply an algorithm and a set of heuristics, described briefly here (Fig. 2; Methods). We adapted sparseMEM (Khan et al. 2009), a program that uses suffix arrays to quickly determine all maximal almost-unique matches (or MAMs) between an NGS read and the reference genome. We use a heuristic to identify distinct and unambiguous matches (or “maps”) spanned by the read pair. The parameters of the heuristic balance the number of maps per read against the quality of the map assignment, and their optimization is described below. The mappings of a read pair provide a unique signature for each SMASH read, allowing easy identification and removal of PCR duplicates.

Our copy number detection algorithm is based on the distribution of map counts and requires that we first establish bin boundaries that partition the genome. We employ bins of expected uniform density, an idea that we first used in single-cell genomic copy number determination (Navin et al. 2011). Boundaries are chosen such that each bin contains the same expected number of maps when sequencing the reference genome with exhaustive coverage and perfect reads. SMASH and WGS have different distributions of expected map densities due to variation in map lengths. To conservatively judge the performance of SMASH, we selected bin boundaries suitable for WGS and mapped the reads in the optimal mode for WGS data: single-end reads using the first 76 bp (Supplemental Table S1). For each sample, we counted the number of maps within each bin and then normalized bin counts for GC bias by a LOESS fit. We used data from whole blood-derived genomic DNA from two families as well as genomic DNA from SKBR3, a mammary cancer cell line. The families are from the Simons Simplex Collection (SSC), each with data from a mother, father, proband, and an unaffected sibling (Fischbach and Lord 2010).

Both WGS and SMASH have distinct patterns of systematic noise that extend beyond GC normalization. This is evident from the strong correlation between independent samples. Moreover, this systematic noise is trendy, leading to high autocorrelation, and can trigger false-positive calls. We correct for this type of error by reference normalization: choosing one sample as a reference and then dividing all remaining sample data by that reference (Levy et al. 2011). The resulting copy number segmentation typically results in segment means that are low integer fractions, reflecting copy number in the sample. To obtain copy number profiles from the bin count data, we use the standard method of circular binary segmentation (Olshen et al. 2004).

### Optimizing pipeline parameters

To measure performance precisely and choose parameters for pipeline processing, we compared the signal in bins on the X Chromosome to those on autosomes in male subjects. We also calculated (1) the median average deviation (MAD) of normalized bin counts to measure the magnitude of the noise and (2) the autocorrelation as a measure of trendiness in the data, an important risk factor for segmentation error. Signal to noise (“S/N”) is calculated as the difference in the medians of the autosomes and X Chromosome, divided by the square root of the sum of the squares of the MADs. We used these statistics to evaluate reference normalization and mapping algorithms and then to compare WGS to SMASH (Table 1).

**Table 1.**
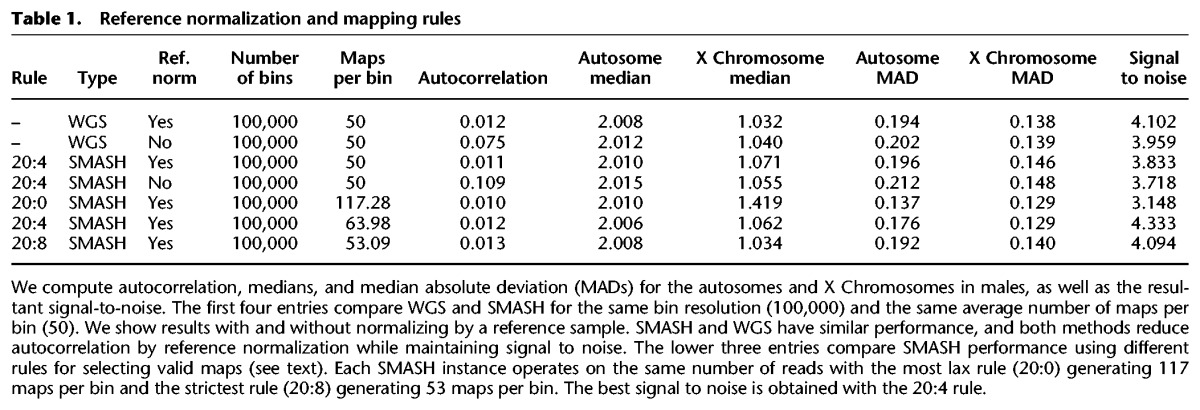
Reference normalization and mapping rules

We first considered the utility of applying reference normalization (Table 1, “ref. norm”). Dividing the GC-adjusted bin ratios by a standard sample bin ratio greatly improved performance for both WGS and SMASH (rows 1–4). Namely, reference normalization decreases autocorrelation up to 10-fold while increasing signal to noise.

Next we established a two-part, two-parameter (L:K) rule for accepting the map of a substring from a SMASH read to the reference genome (Fig. 2A). First, we find all substrings in a read that occur only once in the reference genome and such that the match cannot be extended. These are called “MAMs,” for maximal almost-unique matches (Methods). We demand a minimum match length, L, the first parameter. For the data shown here, L is 20 bp. To avoid false maps that arise by chimerism, we employ a second rule: We demand that a MAM of length M contains a substring of length M−K that maps uniquely to the genome. We examined many combinations of L and K, and we measured their performance on an identical set of SMASH reads, with fixed bin boundaries. We show only the results for rules 20:0, 20:4, and 20:8 (Table 1, rows 5–7). Despite having far fewer maps (“maps per bin”), the 20:4 rule is superior to the 20:0 rule as judged by signal to noise. Many of the 20:0 maps must be false, and we attribute false mapping to chimerism at fragment boundaries. On the other hand, the 20:4 rule is superior to the 20:8 rule as judged by slightly degraded signal to noise, which we attribute to increased sampling error due to reduced coverage. The 20:4 rule utilizes ∼75% of the sequenced bases. If we exclude bases at the end of a read where fragments are typically too short to map, the 20:4 rule uses 85%–88% of bases sequenced (Supplemental Table S2). We employ the 20:4 rule throughout.

### Comparing WGS to SMASH profiles under optimized pipeline parameters

We compared the performance of WGS and SMASH as described above. We consider different total numbers of bins (from 50,000 to 500,000) and different mean numbers of maps per bin (20, 50, and 100), collecting statistics for signal to noise and autocorrelation. Both WGS and SMASH have very similar performance characteristics (Table 2). WGS, map for map, slightly outperforms SMASH. When we choose bin boundaries such that the reference sample has the same number of maps in each bin, the signal-to-noise ratio improved for both SMASH and WGS, and the difference between them narrowed substantially (Supplemental Table S3).

**Table 2.**
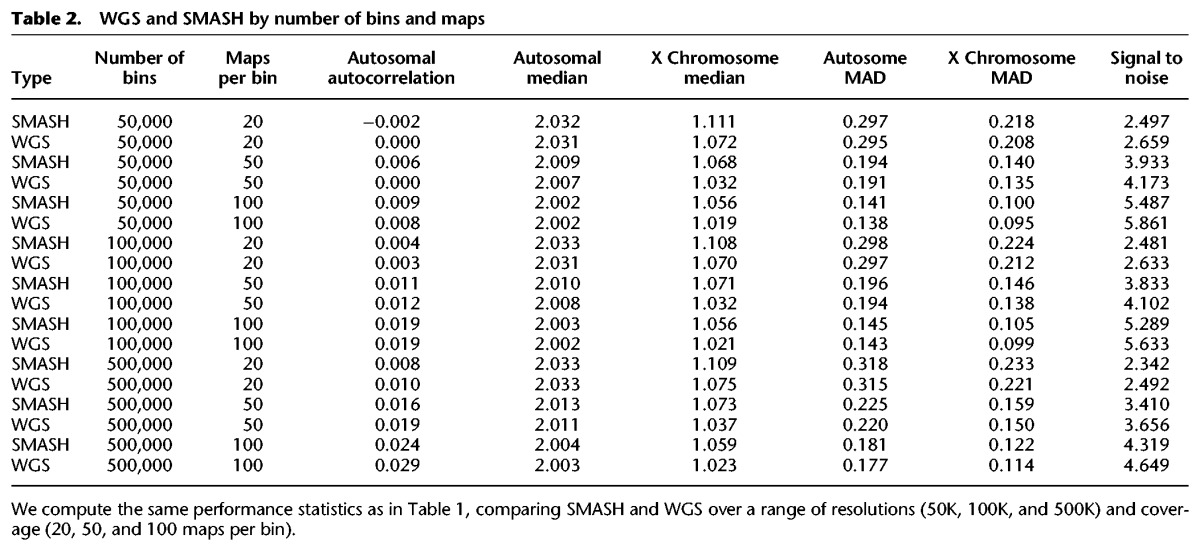
WGS and SMASH by number of bins and maps

As the number of bins increases, the signal to noise diminishes: for SMASH it decreases from 5.6 at 50K bins to 4.0 at 500K bins. Similar degradation of signal occurs for WGS. We hypothesized that this was the result of using the same total number of reference maps for normalization, independent of the number of bins. Therefore, as the number of bins increases, the number of reference maps per bin diminishes, increasing the variance of the normalized ratio. To test if this was the cause, we performed reference normalization—this time matching the total number of reference maps to the total number of sample maps. When we did this, there was virtually no degradation of signal to noise as the bin number increased (Supplemental Table S4).

**Figure 3.**
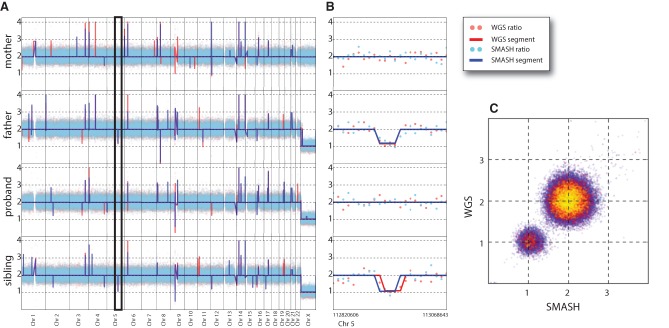
SMASH and WGS copy number profiles for an SSC quad. (*A*) The whole-genome view (autosomes and X Chromosomes) for the four members of a family is shown. Red and blue dots indicate the reference and GC normalized ratio values for WGS and SMASH, respectively. Similarly, red and blue lines represent the copy number segmentation by CBS (circular binary segmentation) for WGS and SMASH. (*B*) A deletion on Chromosome 5 is highlighted (expanded section demarcated in *A*). The deletion, identified by both methods, occurs in the father and is transmitted to the unaffected sibling. (*C*) The bin-for-bin comparison of the normalized ratio values of the father from WGS and SMASH is illustrated. Red and yellow points show increasingly sparse subsamples of the data points.

**Figure 4.**
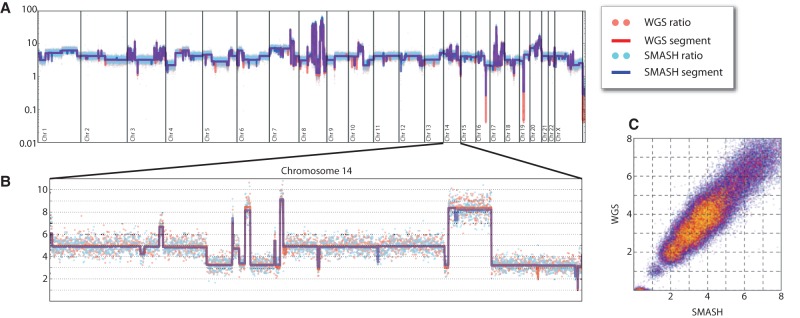
SMASH and WGS copy number profiles for SKBR3. (*A*) The complex copy number pattern within the SKBR3 cell line is shown in whole-genome view. Copy number is indicated on a log scale. The red and blue dots show the GC-normalized ratio values for WGS and SMASH, respectively, while the red and blue lines show the copy number segmentation. (*B*) Chromosome 14 is shown in an expanded view with a linear scale. There is strong agreement between WGS and SMASH in the integer copy number state segmentations and dispersion about the segment mean. (*C*) A bin-for-bin comparison of the normalized ratio values from WGS (*y*-axis) and SMASH (*x*-axis) is displayed as a scatter plot. The red and yellow points show increasingly sparse subsamples of the data points to illustrate density.

Finally, we compared the actual profiles of samples using SMASH and WGS. We used bins optimized for WGS and the map selection rules discussed earlier. We analyzed genomic DNA from two families using reference normalization (Fig. 3) and one cancer cell line without reference normalization (Fig. 4). For comparison, we sampled an equal number of maps from both WGS and SMASH. Across all scales of genome resolution—whether looking at normalized bin counts or segmented data—the profiles from the two methods appear very similar. In both figures, we show 10 million maps distributed into 100,000 bins. Parental transmission patterns appeared largely Mendelian (Fig. 3A). This is illustrated clearly in Figure 3B, which zooms in to show the transmission of a deletion from the father to an unaffected sibling. Whereas the global segmentation patterns generated by SMASH and WGS are not completely identical, much of the variation has to do with segmentation itself. When we look at bin concordance, WGS and SMASH are exceedingly similar (Fig. 3C).

Both WGS and SMASH yielded approximately the same integer-valued copy number profile for the cancer cell line SKBR3 (Fig. 4A). To illustrate the concordance between the data, we zoom in to a single chromosome with extensive genomic copy number variation (Fig. 4B). Again, the bin-for-bin LOESS-adjusted ratios are largely concordant (Fig. 4C). A detailed segment-by-segment comparison of WGS and SMASH across these profiles is provided in the Supplemental Material (Supplemental Table S5). The correlation coefficient between SMASH and WGS is in excess of 0.95 after trimming high copy number outlier segments (<0.01% of the data).

### An alternate fragmentation protocol for SMASH

The initial SMASH protocol combined sonication and RE digestion. We attempted to devise a simpler protocol to yield a tighter fragment length distribution and increase the randomness of SMASH maps. For this purpose, we digested genomic DNA with dsDNA fragmentase (NEB), a combination of enzymes that randomly generates nicks on dsDNA and then cuts the DNA strand opposite the nick, producing dsDNA breaks (Supplemental Methods). Using recommended conditions, we readily obtained fragment lengths with a tighter size distribution and somewhat shorter lengths than those obtained by sonication and RE cleavage. We could readily ligate the fragments and size-select chimeras to an optimal length for sequencing (Supplemental Fig. S3). We then compared this protocol (“SMASH2”) to our initial protocol on genomic DNA from the cancer cell line SKBR3, without normalization. The copy number profiles generated by the two methods were virtually identical (Supplemental Fig. S4). The average number of maps increases by more than one per read pair with the SMASH2 method (Illumina NextSeq 500, 2 × 150 bp run mode). We believe the improvement is due to more precise sizing achievable in this protocol.

## Discussion

SMASH has one clear advantage over standard WGS for obtaining copy number information: each sequence read is packed with multiple independent mappings, increasing the information density per read and thereby lowering the cost per sample. Map for map, SMASH is comparable in quality to WGS with respect to copy number profiling. While employing longer reads in WGS may yield additional structural information, such as the breakpoints of copy number events, small-scale indels and point mutations, the identification of these elements requires orders of magnitude more coverage than what is needed for copy number analysis. For detecting CNVs several kb and larger, the choice should be driven by cost.

Our observations on WGS read length and copy number measurement suggest that the most cost-efficient WGS sequencing mode for CNVs would employ shorter reads than SMASH. Based on current systems in widespread usage (e.g., the Illumina HiSeq 2500) on which samples can be multiplexed, the most cost-effective mode currently available for effective genomic mapping of WGS reads is 1 × 36 bp in high-output run mode (Supplemental Table S6). For SMASH, the most optimal mode on this instrument is the 2 × 125 bp high-output format. The relative costs of producing “standard” WGS and SMASH libraries vary by <10% (SMASH2 protocol). Based on these criteria and our sequencing costs (which would be expected to scale similarly at larger genome centers), SMASH can produce copy number data at 20-kb resolution for ∼55% of the total cost of WGS, and <50% at higher resolution (Supplemental Table S6). With longer read lengths such as 2 × 150 bp available on newer production-scale instruments such as the HiSeq 3000/4000, this cost advantage is likely to grow further.

We have invested significant effort in optimizing the design of the SMASH protocol and algorithms. These include rules for mapping SMASH reads, normalizing bin counting, and methods for fragmentation. With the simplified SMASH2 protocol, we obtain more maps per read pair with comparable resolution on a map-for-map basis. Further improvements are possible, especially in algorithmic development. For example, in this iteration of SMASH, we have ignored fragments that map to duplicated regions of the genome. As these areas will be prone to copy number variation, future versions should include such maps in the analysis.

For most of the analysis of maps, we used bin boundaries determined for WGS so that we could directly compare SMASH to WGS under conditions favoring the latter. However, we have shown that the optimal bin boundaries are those derived empirically to yield uniform map counts (Supplemental Table S4). From such work, it is clear that increasing the coverage of the reference sample used for normalization will improve signal-to-noise ratios for all samples. We do not yet see a lower limit to the level of resolution that can be obtained.

The protocols and sequencing depths demonstrated in this paper are sufficient to identify CNVs of >10 kb, generating higher resolution profiles than the arrayCGH platforms currently in common use. Further, while the SMASH method at present requires 200 ng of genomic DNA, more than is needed for WGS, this is less than the requirements for arrayCGH. As such, the SMASH protocol presented here is quite competitive with the current standard of care for pediatric genetics.

For a given sequencing instrument, the resolution of SMASH (like WGS) is unlimited and scales with the number of reads. But in the future, advances in sequencing technology that reduce unit cost per base pair will likely be driven by increasing read lengths. For copy number inference, this means a continued decline in the number of maps per base sequenced from WGS. However, SMASH, even with existing sequencers, can yield four to six times as many maps per read as standard WGS. Thus, SMASH can reduce the costs of testing in prenatal, pediatric, and cancer genetics, allowing more patients to be tested at lower cost.

## Methods

### DNA materials

Genomic DNA used in this study was from two sources. The first source was SKBR3, a human breast cancer cell line. The second source was whole blood-derived DNA from two families of the Simons Simplex Collection (SSC).

### SMASH protocol

The amount of genomic DNA required for SMASH is variable. We tested three different genomic DNA inputs—200 ng, 500 ng, and 1 µg—and successfully constructed high-quality libraries for all three conditions. In this study, we used 1 µg of DNA as starting material from all the samples. DNA was diluted in 1× Tris buffer (10 mM Tris-Cl at pH 8.5) to a final volume of 75 µL and was transferred to microtubes (Covaris). The Covaris E210 AFA instrument (Covaris) was used to shear genomic DNA to ∼100-bp fragments according to the manufacturer's manual. DNA fragments were further cut by CviKI-1 (NEB) and NlaIII (NEB) in 1× CutSmart buffer in a final volume of 90 µL, which was incubated at 37°C for 1 h. After enzymatic digestion, the solution volume was reduced to ∼30 µL by Savant SpeedVac (Thermo Scientific). DNA fragments >100 bp were removed as follows: adding 2.5× volume of AMPure XP beads (Beckman Coulter), mixing well, incubating at room temperature (RT) for 5 min, and collecting the supernatant. The supernatant was then purified by QIAquick nucleotide removal kit (Qiagen), following the manufacturer's instructions. DNA fragments were eluted in 30 µL H_2_O. The average length of DNA fragments was 40–50 bp as determined by the Bioanalyzer 2100 (Agilent Technologies). These DNA fragments were end-repaired by T4 DNA polymerase (NEB), DNA polymerase I (large Klenow fragment, NEB), and T4 polynucleotide kinase (NEB) at RT for 30 min. The polished DNA fragments were purified by QIAquick nucleotide removal kit (Qiagen) and eluted in 30 µL H_2_O. The short DNA fragments were randomly ligated to form longer stretches of chimeric DNA with the quick ligation kit (NEB) at RT for 15 min. The long DNA chimeras were purified using 1.6× AMPure XP beads and end-repaired as earlier. A single adenine nucleotide was added to the 3′ ends of the polished DNA fragments by Klenow fragment (3′→5′ exo, NEB) at 37°C for 30 min. After purification by 1.6× AMPure XP beads, barcoded sequencing adapters (Iossifov et al. 2012) were ligated to the DNA fragments by quick ligation. This allowed us to multiplex samples on sequencing lanes. DNA fragments were again purified by 1.6× AMPure XP beads and eluted in 50 µL H_2_O. This size-selection step was carried out to enrich for DNA fragments within the ideal Illumina sequencing length range of 300–700 bp. First, 0.6× (30 µL) AMPure XP beads was added into 50 µL of purified DNA. After incubation at RT for 5 min, supernatant was collected. Eight microliters (0.16× the original 50 µL) of AMPure XP beads was added and mixed well with the supernatant. This mixture was incubated at RT for 5 min. After two washes with 180 µL of 80% ethanol, DNA fragments were eluted in 30 µL H_2_O. The final eight cycles of PCR amplification were carried out on this DNA using Illumina sequencing adapters in 1× Phusion high-fidelity PCR master mix with HF buffer (NEB). DNA libraries were quantitated on the Bioanalyzer and diluted to a concentration of 10 nM. Sequencing was performed on the HiSeq 2000 (2 × 101 bp run mode, Illumina) for libraries prepared from SSC families and on the NextSeq 500 (2 × 150 bp run mode, Illumina) for libraries prepared from the SKBR3 cell line.

### Determining maps

We mapped WGS and SMASH data to the GATK b37 genome. For WGS, we clipped read 1 to 76 bp, mapped it using Bowtie (Langmead et al. 2009), and then filtered duplicate reads using SAMtools (Li et al. 2009). For SMASH (after the mapping procedure described below), we used the multiple-MAM signature of each read pair to filter duplicates. For both methods, we established bins and counted unique mappings for Chromosomes 1–22, X, and Y only.

To prepare for mapping SMASH data, we modified the sparseMEM package (Khan et al. 2009) to increase the maximum genome size from 2.147 × 10^9^ bases to an essentially unlimited value, and we removed the sparse functionality to increase program speed and decrease complexity. We added features (1) to save the various suffix array index structures to disk; (2) to read suffix array index structures in for subsequent runs using memory-mapping; (3) to distribute reads to the parallel query threads to avoid multiple parsing of the input; and (4) to read several query files in parallel. We also added options to read input data from FASTQ and SAM files, to output mappings and nonmapping reads in SAM and custom binary formats, and to simultaneously map to the genome and its reverse complement to avoid a maximal exact match (MEM) pruning step. The resulting software package is called longMEM for its ability to handle longer genomes. For 10 million read pairs (2 × 150 bp), at peak use, the longMEM mapper employed 117 GB of RAM and 12 threads for parallel processing on our computational cluster (Intel Xeon 2690 CPUs). Mapping and map selection required 40 min. For subsequent processing steps including binning, only a single thread was necessary, and the task was completed in 2 min.

Using longMEM, we searched for MAMs, which are maximally extended subsequences in query reads that match uniquely within the reference and its reverse complement but may be repeated in the query. For query reads of length Q and a reference of length R, we find all MAMs in the query in O{Q × [Q + log(R)]} time using the reference, the suffix array, its inverse, and an LCP (longest common prefix) table.

Most tags comprising SMASH reads result in MAMs that are suitable for copy number analysis. The exceptions are fragments that are not present in the reference due to blocking read errors or mutation, as well as those that are too short to be uniquely mapped to their origin. In addition to acceptable MAMs, junctions between adjacent tags in SMASH sometimes result in false MAMs. To filter spurious MAMs, we apply a two-parameter filtering rule (L:K). A MAM passes the (L:K) filter if it (1) is at least L base pairs in length and (2) would still map uniquely to the genome if reduced in length by K base pairs from either end. An additional filter ensures that read pairs contain no MAMs within 10 kb of another. This avoids double counting of fragments containing indels or SNPs, as well as fragments that span both ends of the read pair.

### Binning, normalization, copy number, and signal to noise

We divided the autosomes and the X and Y Chromosomes into 50,000, 100,000, and 500,000 WGS-optimized bins by mapping every 50-mer in the reference with Bowtie and adjusting bin boundaries so that each bin had the same number of uniquely mapped reads assigned to it (±1).

We assigned an equal number of mappings from SSC WGS and SMASH data to bins and added one count to each total. We normalized counts to set the mean of all autosome bins to one and then performed LOESS on the normalized autosome to correct for GC content. After bin-wise summation across samples, we selected “bad” bins based on upward copy number deviation from the chromosome median exceeding a MAD-based limit using a Bonferroni-corrected *P*-value of 0.05.

We sampled SSC and SKBR3 mappings at 20, 50, 100, and up to 1000 per bin (when available) and assigned them, excluding bins marked as “bad.” We performed bin-wise normalization of sample counts using an unrelated male reference sample at high coverage. We normalized and GC-corrected the ratio data and then segmented the result using circular binary segmentation (Olshen et al. 2004) with the minimum segment length and alpha parameters set to three and 0.02, respectively. We adjusted the segmented profiles by varying the overall scale and offset within expected bounds to find the best quantal fit.

We defined SSC sample signal to noise for SMASH and WGS as the autosome median minus the X Chromosome median unquantized ratio, divided by its measured MAD-based noise for male samples using a female reference sample (when performing reference normalization).

## Data access

All data sets from this study have been submitted to the NCBI Sequence Read Archive (SRA; http://www.ncbi.nlm.nih.gov/sra/) under accession number SRP069815. Source code, including longMEM, SMASH mapping heuristics, bin boundaries, map counting, and copy number profiling, is available for download at https://github.com/docpaa/smash-paper and in the Supplemental Material.
